# A new spring for titin

**DOI:** 10.1113/JP279314

**Published:** 2020-01-09

**Authors:** Elisabetta Brunello, Luca Fusi

**Affiliations:** ^1^ Randall Centre for Cell and Molecular Biophysics and BHF Centre of Research Excellence King's College London London UK

**Keywords:** muscle contraction, sarcomere, skeletal muscle fibre, titin filaments

Titin, the third filament of striated muscle, has been largely neglected in mechanical models of the sarcomere. The titin filament spans the entire length of the half‐sarcomere forming extensible links in the I‐band of the sarcomere between the tips of the thick filaments and the Z‐band. These titin links act as molecular springs that account for the passive force response to stretch of a resting muscle (Granzier & Wang, 1993). However, the stiffness of the titin spring, determined from the relationship between passive force and sarcomere length, is much lower than that of the acto‐myosin springs, suggesting that its contribution to the mechanical properties of the active sarcomere is negligible.

Recent *in vitro* studies have challenged this conclusion, proposing the idea that folding of immunoglobulin(Ig)‐like domains in the I‐band titin might contribute to the mechanical work done by muscle during contraction (Rivas‐Pardo *et al*. 2016). However, the power generated by titin folding would be ∼1000 times smaller than that generated by the myosin motors, and therefore the role of titin as a force generator in the active muscle seems unlikely (Bianco *et al*. 2016). Another recent study showed that titin‐based passive stress can activate the thick filament in skeletal muscle, independently of calcium, suggesting a possible role of titin in the regulation of muscle contractility, likely mediated by the mechanosensing signalling pathway in the myosin filament (Fusi *et al*. 2016). Therefore, a new role of titin in muscle contraction is emerging, but the mechanical function of the titin filament *in situ* in the active sarcomere remains to be clarified.

In this issue of *The Journal of Physiology*, Powers *et al*. (2020) provide a quantitative analysis of the mechanical properties of the I‐band titin spring in intact muscle fibres of the frog, in which the physiological arrangement of the titin filaments in the myofilament lattice is preserved. Measuring the elasticity of this spring in the active sarcomere is technically challenging, as the sarcomere stiffness in a contracting muscle is dominated by the stiffness of the myosin motors attached to actin. Recently, Lombardi's group has developed a new strategy to isolate the contribution by additional springs to sarcomere elasticity during contraction. Their approach uses small and rapid length oscillations applied to electrically stimulated muscle fibres to measure the time course of the sarcomere stiffness during an isometric contraction. In particular, the stiffness measurements related to the early phases of force generation after stimulus, when the number of actin‐attached myosin motors is low, allow the stiffness of the additional spring to be determined *in situ* during contraction. Powers *et al*. performed stiffness measurements during contractions in the sarcomere length range from 2.15 to 3.10 µm and determined the stiffness of titin in the active sarcomere by fitting the data to a new mechanical model of the sarcomere that includes an I‐band spring. Interestingly, they found that the contribution of titin to the stiffness of the sarcomere appears only at sarcomere lengths >2.5 µm and that titin stiffness during contraction is ∼100 times higher than that previously determined from the passive force–sarcomere length relationship. Such a large discrepancy between estimates of titin stiffness can be explained by the fact that the fast mechanical method used by Powers *et al*. allows the undamped elasticity of the spring to be measured, independently of the visco‐elastic relaxation processes that occur in the titin molecule on a slower time scale and affect the steady‐state relationship between passive tension and sarcomere length.

The other key finding is that the titin elasticity remains constant in the sarcomere length range from 2.7 to 3.1 µm. The authors suggest that this could be explained by an adaptive mechanism that is able to maintain the length of the titin spring constant in response to an increase in the width of the I‐band at longer sarcomere lengths. On the basis of the mechanical properties of the Ig domains in the I‐band titin (Rivas‐Pardo *et al*. 2016) the authors speculate that the titin spring, mainly accounted for by the PEVK domain, could maintain its length and stiffness constant thanks to the unfolding of the flanking Ig domains at longer sarcomere length.

Although the molecular mechanism of the I‐band spring tunability remains to be elucidated, the results by Powers *et al*. suggest that titin, thanks to its dynamic stiffness, might play a fundamental and previously unrecognized role in muscle contraction, by allowing weaker half‐sarcomeres to equilibrate with in‐series stronger half‐sarcomeres, thus preventing the development of sarcomere length inhomogeneity during high‐load contractions. The results presented by Powers *et al*. shed new light on the function of titin *in situ* in the active sarcomere and open new questions about the mechanical properties of the I‐band springs of different titin isoforms and their functional impact on the contractility of skeletal and cardiac muscle.

## Additional information

### Competing interests

None declared.

### Author contributions

Both authors have approved the final version of the manuscript and agree to be accountable for all aspects of the work in ensuring that questions related to the accuracy or integrity of any part of the work are appropriately investigated and resolved. All persons designated as authors qualify for authorship, and all those who qualify for authorship are listed.

### Funding

E.B. was funded by a British Heart Foundation Intermediate Basic Science Research Fellowship (FS/17/3/32604). L.F. was funded by a Sir Henry Dale Fellowship awarded by the Wellcome Trust and the Royal Society (210464/Z/18/Z).
